# The ambiguous feeling between “mine” and “not-mine” measured by integrated information theory during rubber hand illusion

**DOI:** 10.1038/s41598-022-22927-1

**Published:** 2022-10-26

**Authors:** Takayuki Niizato, Yuta Nishiyama, Kotaro Sakamoto, Takumi Kazama, Tatsuya Okabayashi, Taiki Yamaguchi

**Affiliations:** 1 Faculty of Engineering, Information and Systems, University of Tsukuba, Tsukuba, Japan; 2 Department of Information and Management Systems Engineering, Nagaoka University of Technology, Niigata, Japan; 3 The Institute of Statistical Mathematics, Research Center for Statistical Machine Learning, Tokyo, Japan

**Keywords:** Applied mathematics, Consciousness, Sensory processing, Human behaviour

## Abstract

Human body awareness is adaptive to context changes. The illusory sense of body ownership has been studied since the publication of the rubber hand illusion, where ambiguous body ownership feeling was first defined. Phenomenologically, the ambiguous body ownership is attributed to a conflict between feeling and judgement: it characterises a discrepancy between first- and third-person processes. Although Bayesian inference can explain this malleability of body image, it still fails to relate the subjective feeling to physiological data. This study attempts to explain subjective experience during rubber hand illusions by using integrated information theory (IIT). The integrated information $\Phi$ in IIT measures the difference between the whole system and its subsystems. By analysing seven different time-series of physiological data representing a small body–brain system, we demonstrate that the integrity of the whole system during the illusion decreases, while the integrity of its subsystems increases. These general tendencies agree with many brain-image analyses and subjective reports; furthermore, we found that subjective ratings as ambiguous body ownership were associated with $\Phi$. Our result suggests that IIT can explain the general tendency of the sense of ownership illusions and individual differences in subjective experience during the illusions.

## Introduction

The malleability of human body image is a manifestation of the flexible perceptual system. This malleability has been demonstrated in several experiments where the body awareness is altered by multisensory stimuli. One famous example is the rubber hand illusion (RHI) introduced by Botvinick and Cohen^1^, where a subject observes a brush stroking a dummy hand while the subject’s real hand, which is hidden behind a partition, is also stroked. Synchronised visuo-tactile stimuli lead to an illusory sense of ownership of the dummy hand. The body ownership of the dummy body parts can be extended to the whole body. For instance, Lenggenhager et al. showed that a person who observed a virtual body being touched while they were simultaneously also being touched experienced an out-of-body experience^2^. Interestingly, the stimulation of specific brain regions, specifically the temporo-parietal junction area, can evoke a similar experience^3^. This region is known to work in consonance with multiple body sensations^4^. Yet despite numerous studies conducted in this regard, a comprehensive understanding of the sense of ownership is still lacking. Recent phenomenological interpretations provide a concise scheme for understanding the origin of body ownership and agency^5–8^. Ataria summarised the type of feeling-judgement conflict to clarify the various states of body ownership^9^. Phenomenologically, feelings involve closed sensor-motor loops, whereas judgements are more abstract reasoning processes. In summary, feelings provide a first-person perspective, i.e., a non-conceptual, pre-reflective, bottom-up process; by contrast, judgement gives a third-person perspective, i.e., an observational, reflective, top-down process. According to this argument, during an RHI, subjects feel the dummy hand as their hand but never admit (or judge) it as their hand. This conflict between feeling (i.e., ‘this is my hand’) and judgement (i.e.,‘this is not my hand’) causes ambiguous body ownership (ABO) rather than a specific sense of body ownership^7^. The fact that the results of the two processes are not necessarily consistent motivated us to consider that malleability of body image originates during the mismatched feeling-judgement process.

The free-energy principle (FEP; or Bayesian inference) is a promising theory for understanding the flexibility of our perceptual system^10^. According to the FEP, we can change our beliefs whenever confronted with unexpected situations (i.e., high saliency). The perceptual system updates prior hypotheses with the preferable posterior hypotheses. The FEP suggests that appropriately adjusting the hypotheses in response to the environment corresponds to the malleability of body image. The RHI is, therefore, related to the corruption of sensorimotor predictive cycles. Phenomenologically, this sensor-motor prediction error indicates a corruption of the feeling system. Most studies do not proceed further than this interpretation of corruption at the feeling level; however, as discussed earlier, the corruption of the feeling system does not provide an adequate explanation for ABO because we must also consider the top-down judgement level.

Tsakiris et al. investigated the interaction between bottom-up and top-down processes through interoceptive and exteroceptive perceptions^11–13^. They showed that the intensity of RHI is due to the mismatch between interoceptive and exteroceptive predictions. In their experiments, subjects who predicted their heartbeats more precisely experienced less illusion of ownership during the RHI than subjects who did so less precisely^11^. They speculated the existence of an interdependent relationship between interoceptive and exteroceptive predictions, i.e., that high interoceptive saliency suppresses exteroceptive saliency, resulting in ABO^14^.

Although Tsakiris^14^ proposed a remarkable relationship between RHI subjectivity and FEP, FEP per se is still fraught with a difficulty: specifically, it assumes that judgement is a process for selecting the highest saliency among possible saliencies. As discussed in Ransom et al.^15^, these saliency selections cannot explain human affective behaviour for low saliency. This limitation implies that the saliency-driven explanation is insufficient for human experience. If we accept the saliency selection, then we agree that there is no conflict between feeling and judgement, because the judgement system mechanically picks up the highest saliency. In this case, the intensity of RHI is ascribed only to the feeling system. If we do not accept the saliency selection, then we agree that the sensor-motor cycle holds both feeling and judgement. In this case, we neglect the essential difference between feeling and judgement owing to the compression of two different systems into one sensor-motor system.

Integrated information theory (IIT) has been proposed to mathematically define human consciousness^16–19^. The basic concept of IIT is summarised in the proposition that the degree of consciousness can be measured as the difference between the whole system and the sum of its parts; that is, the irreducibility of the whole system to its parts is the key to understanding the experience of consciousness^16,17^. Notably, IIT also considers both feeling and judgement in the perceptual process. Feeling corresponds to the local process and represents the information process of the subsystem (i.e., the subsystems of the system). By contrast, judgement corresponds to the global process and represents the information process of the whole system. Importantly, IIT rejects any external evaluations as an explanation for consciousness. Therefore, there is no superiority between feeling and judgement. In this sense, judgement maintains a parallel relationship with feeling in IIT, in contrast to FEP. The difference between feeling and judgement is revealed only by subjective experience. Thus, IIT can mathematically evaluate body malleability induced by the conflict between feeling and judgement.

In a recent study that took physiological measurements during RHI, autonomic nervous activity mediating interoception did not change under an altered body–representation^20^. Thus, merely measuring autonomic activity may not be enough to reveal effects of both feeling and judgement on subjective experience during RHI. Instead, it would be effective to measure autonomic activity with central nervous activity mediating exteroception. We thus measured multiple physiological activities and examined its relationship with subjective experience using IIT. Our measurements took in both brain activities and internal bodily states, whereas IIT is usually applied only to the complex neural networks in the brain. We measured seven types of physiological data from the body (respiration, heartbeat, and skin conductance) and brain (electroencephalogram [EEG] with electrodes on midline frontal [Fz], vertex [Cz], midline parietal [Pz], and midline occipital [Oz] regions). Notably, affective stimuli such as apparent threat to the rubber hand evoking transient physiological responses^21–26^ were not applied because we were interested in a wide range of physiological changes over time associated with the illusory body ownership during the classical RHI. Hence, we focused on these data to explore whether the interaction between interoceptive (body-related responses) and exteroceptive (part of brain activity) processes reflected the subjective experience of ABO during RHI, as highlighted by Tsakiris^14^. We predicted that the degree of conflict between body and brain, rather than that of the whole brain process, is enough to estimate experiences during RHI.

## Results

### Minimum information partition (MIP)

We examined the average tendency of MIP during the experiments. MIP gives two measures for estimating the system’s state: (i) MIP cut, which divides the two subsystems by the weakest information flow; and (ii) $\Phi _{\mathrm {MIP}}$, which is the degree of information loss by the MIP cut. $\Phi _{\mathrm {MIP}}$ is positive by definition; otherwise (i.e., if it were equal to 0), the two subsystems would be completely independent.

This study used seven datasets: respiration (RES), heartbeat (ECG), and skin conductance (EDA) for body, and EEG from midline frontal (Fz), vertex (Cz), midline parietal (Pz), and midline occipital (Oz) regions for brain, for IIT analysis. We considered that these body–brain signals work as a system ($S$ = {Res, ECG, EDA, Fz, Cz, Pz, Oz}: seven node’s all connected network). If the rubber hand stimulus causes a difference in the system, the relation among these seven datasets will change. This difference also reflects the MIP information.

**Figure 1:**
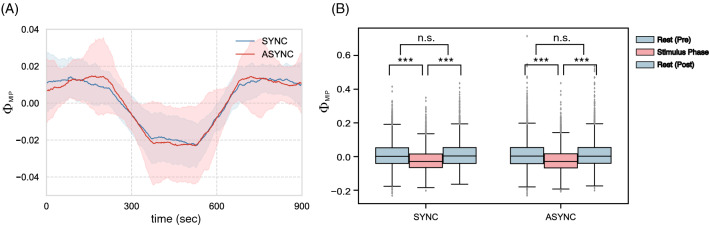
(**A**) Time series of mean $$\Phi _{\mathrm {MIP}}$$ for both conditions (blue: SYNC, red: ASYNC). The stimulus phase covers $$300{-}600$$ s. The other regions indicate resting states (no stimulus). The computational time frame for IIT is 250 (2 s). A moving-average (sample length 150 steps) was calculated in the time series to make temporal changes of values easy to see (Niigata) $$\Phi _{\mathrm {MIP}}$$ of SYNC and ASYNC values drops significantly compared with each rest state ($$*p < 0.05$$, $$**p < 0.01$$, $$***p < 0.005$$).

Figure 1A shows the time series of the mean $\Phi _{\mathrm {MIP}}$ for all 22 subjects. The $\Phi _{\mathrm {MIP}}$ drops at the stimulus phase in both synchronous (SYNC) and asynchronous (ASYNC) conditions (Fig. 1B). This decrease suggests that connections between subsystems become weaker than during pre- and post-stimulus phases. This result is interesting because similar changes occur in both conditions.

**Figure 2:**
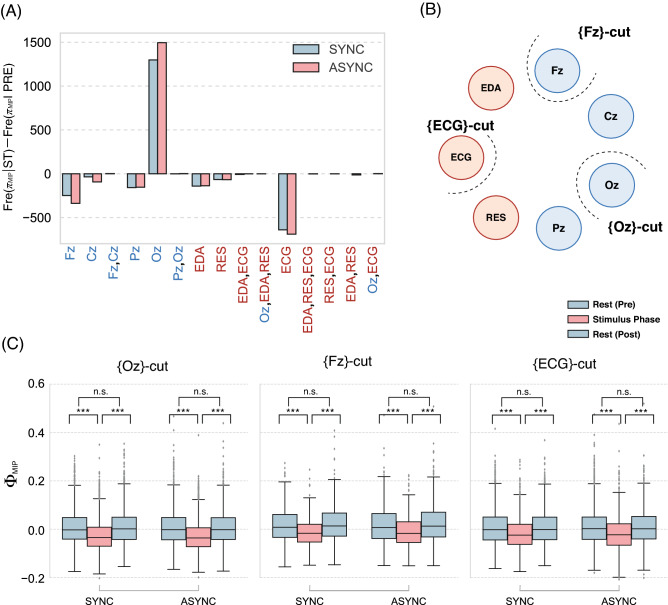
(**A**) Differences in the $$\pi _{\mathrm {MIP}}$$ frequencies of pre- and intra-stimuli: Fr$$(\pi _{\mathrm {MIP}}|\mathrm {ST})-$$Fr$$(\pi _{\mathrm {MIP}}|\mathrm {PRE})$$. The computational time frame for IIT is 250 (1 s). The positive values indicate that a certain type of $$\pi _{\mathrm {MIP}}$$ increases during the stimulus. Blue (red) bars show the frequency differences in the synchronous (asynchronous) condition. Blue (red) letters show data from the brain (body) system. (**B**) Image of MIP-cut location of the system *S*. {Oz}-, {Fz}-, {ECG}-cut are the subsets with the three largest differences shown in (**A**). (**C**) $$\Phi _{\mathrm {MIP}}$$ of each MIP-cut location. In all cases, $$\Phi _{\mathrm {MIP}}$$ drops during the stimulus ($$*p < 0.05$$, $$**p < 0.01$$, $$***p < 0.005$$). No after-effects were observed (see Tables S2 and S3 for other parameter settings).

Next, we examined the details of the MIP cut. The most remarkable tendency was the cut between {Oz} and {RES, ECG, EDA, Fz, Cz, Pz}. More than 50% of MIP cuts were of this type (Fig. 2A,B); furthermore, during the stimulus phase, the frequency of this cut increased statistically. Although its frequency increased, the $\Phi _{\mathrm {MIP}}$ of the {Oz}-cut decreased. The implication of this tendency is suggestive. It is a well-known fact that the occipital region (Oz) is involved in the visual information process. The high frequency of {Oz}-cut reflects the discrepancy between visual information and tactile information. The low $\Phi _{\mathrm {MIP}}$ during the stimulus means that the visual process in Oz diverges from other information processes (Fig. 2C).

In the remaining patterns, the MIP-cut frequencies decreased after the stimulus was applied (Fig. 2A,B). The decreased frequency of the rest compensates for the increased frequency of the {Oz}-cut. Interestingly, in the first case we observed, the cut was rarely located between the body and brain systems. But in most cases, the cut was located in the brain system. This fact suggests that the body and brain systems are not the weakest links. The second most frequent $\Phi _{\mathrm {MIP}}$ cut is located between {ECG} and {RES, EDA, Fz, Cz, Pz, Oz}. According to this cut, the heartbeat (ECG) merges into the cycle of the brain–body system during the stimulus. Even if {ECG} was selected as the MIP-cut, its integrity decreased during the stimulus. This aspect resonates with Tsakiri’s result^11^.

Finally, we highlight the fact that $\Phi _{\mathrm {MIP}}$ for SYNC and ASYNC conditions during RHI showed a statistical difference between the two conditions observed (Welch’s *t* test: *df* = 13159, t-stat = 2.2561, $p<0.05$). This result seems interesting because half of the subjects in our study reported a change of body ownership during both conditions. Many of the individual differences were perceived not in nature but in degree. This logic also applies to the next subsection.

### Main complexes analysis

We examined the average tendency of the main complexes under SYNC and ASYNC conditions. The concept of the main complex accompanies some other concepts; namely, (i) $\Phi _{\mathrm {MIP}}^{T}$, which is the degree of information loss caused by partitioning the system into independent subsystems; the manner of partitioning system *T* is determined by MIP: $\Phi _{\mathrm {MIP}}^{T}$; (ii) complex *T*, where all subsystems satisfy $\Phi _{\mathrm {MIP}}^{T}$>0 and $\Phi _{\mathrm {MIP}}^{T}>\Phi _{\mathrm {MIP}}^{R}$ for any superset *R* of *T*; and (iii) main complex *M*, where the complex *M* satisfies $\Phi _{\mathrm {MIP}}^{M}>\Phi _{\mathrm {MIP}}^{U}$ for any subset *U* of *M*.

Because $\Phi _{\mathrm {MIP}}$ represents the irreducibility to the system’s parts, the high $\Phi _{\mathrm {MIP}}$ values indicate the system’s integrity. Notably, the system may include several main complexes. For a subsystem to be the main complex, it is sufficient that it does not contain other complexes (Lemma 4 in Kitazono et al.^27^). In our analysis, the body–brain system has up to ten or more main complexes. The integrity of the system can be estimated as the sum of $\Phi _{\mathrm {MIP}^{M}}$ of all main complexes *M* in *S* (denoted $\sum _{M\in \mathcalligra {M}}\Phi _{\mathrm {MIP}}^{M}$ where $\mathcal {M}$ is a set of main complexes of system $S$ = {Res, ECG, EDA, Fz, Cz, Pz, Oz}).

**Figure 3:**
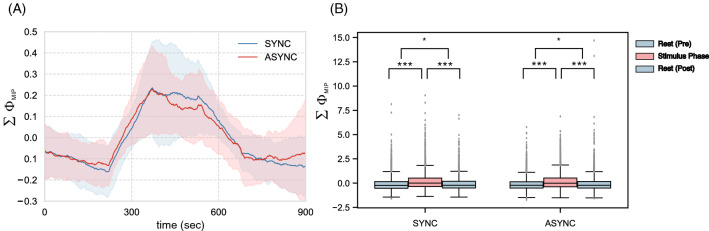
(**A**) Time series of mean $$\sum _{M\in \mathcalligra {M}}\Phi _{\mathrm {MIP}}^{M}$$ in both conditions (blue: SYNC, red: ASYNC). The stimulus phase is $$300 - 600$$ s. The other regions are in resting phase (no stimulus). The computational time frame for IIT is 250 (1 s). Notably, the moving-average (sample length 150 steps) was calculated in the time series to make temporal changes of values easy to see. (**B**) $$\sum _{M\in \mathcalligra {M}}\Phi _{\mathrm {MIP}}^{M}$$ of SYNC and ASYNC values rises significantly compared with each rest phase ($$*p < 0.05$$, $$**p < 0.01$$, $$***p < 0.005$$). In contrast to the MIP analysis, an after-effect was observed in both conditions.

Figure 3A shows that $\sum _{M\in \mathcalligra {M}}\Phi _{\mathrm {MIP}}^{M}$ increases only in the stimulus phase. In contrast to the decreasing $\Phi _{\mathrm {MIP}}^{S}$ for the whole system *S*, the integrities of the particular subsystems increased (Fig. 3B). While the whole system splits into relatively independent parts, specific subsystems compensate by their integrity. This tendency can also be observed in the number of complexes. The number of irreducible subsystems (parts) increased during the stimulus phase (Fig. S4). Furthermore, in contrast to the $\Phi _{\mathrm {MIP}}^{S}$, an after-effect was observed in the main complex. This means that fluctuations in the system integrities remained even after the stimulus had ended.

**Figure 4:**
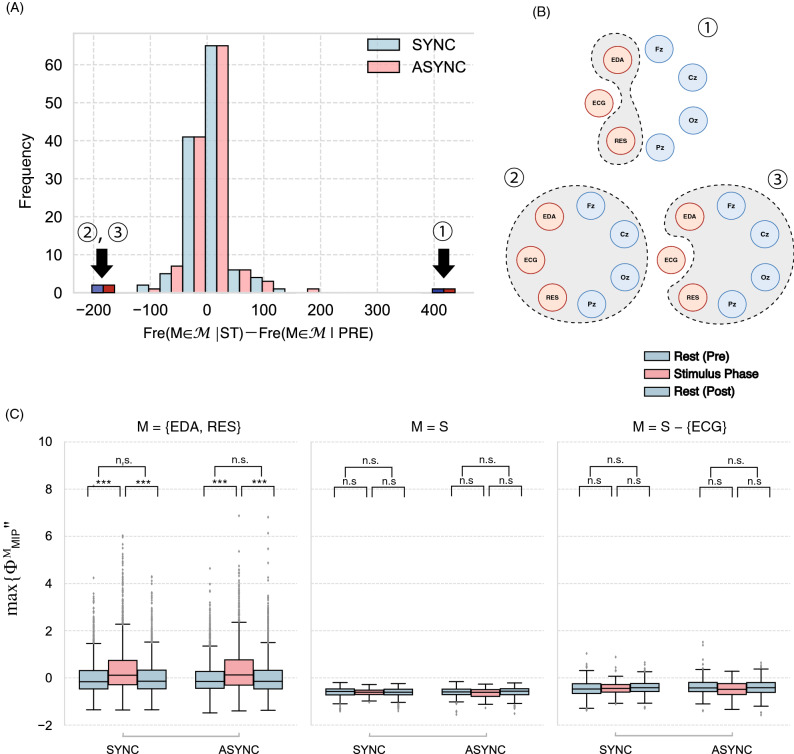
(**A**) Histogram of the difference in the $$\max _{M\in \mathcalligra {M}}\{\Phi _{\mathrm {MIP}}^{M}\}$$ frequency for pre- and intra-stimuli: Fr$$(M\in \mathcalligra {M}|\mathrm {ST})-$$Fr$$(M\in \mathcalligra {M}|\mathrm {PRE})$$. Because many main complexes exist, we analysed only the outliers (indexed 1, 2, 3). Blue (red) bars show the frequency in the synchronous (asynchronous) condition. (**B**) Image of the main complex for the system *S*. {EDA, RES}, *S*, and *S*-{ECG} are the three greatest differences. (**C**) $$\max _{M\in \mathcalligra {M}}\{\Phi _{\mathrm {MIP}}^{M}\}$$ for each main complex. Only for $$M$$ = {EDA, RES} does $$\Phi _{\mathrm {MIP}}$$ rises significantly during the stimulus. The other two values are negative throughout the three phases ($$*p < 0.05$$, $$**p < 0.01$$, $$***p < 0.005$$. See Tables S4 and S5 for other parameter settings).

The content of the most vital main complex (i.e., $\max _{M\in \mathcalligra {M}}\{\Phi _{\mathrm {MIP}}^{M}\}$: the main complex *M* with the highest $\Phi _{\mathrm {MIP}}^{M}$) is more suggestive. Figure 4A shows that the frequency of the main complex containing all elements (or $\Phi _{\mathrm {MIP}}^{S}$) dropped during the stimulus phase. Here, the value of $\Phi _{\mathrm {MIP}}^{S}$ indicates that the body–brain unit itself is irreducible to its parts. Metaphorically, the oneness of the body–brain system was corrupted during the stimulus. After the stimulus phase, the frequency of this main complex recovered.

The other main complexes compensated for the decreasing frequencies of the complexes *S* (Fig. 4B,C). The main complex {EDA, RES} is the most significant, showing skin conductance and respiration. The $\Phi _{\mathrm {MIP}}^{\mathrm {\{EDA, RES}\}}$ also increased significantly during the stimulus (Fig. 4C). The integrity only involves the body parts. Another high frequent main complex is {Fz, Cz, EDA, RES}, containing {Fz, Cz} pairs. The frequency of this complex increased during the stimulus (the $\Phi _{\mathrm {MIP}}^{\mathrm {\{Fz, Cz, EDA, RES}\}}$ also increased: Welch’s *t* test: *df* = 553, t-stat = − 4.0359, $p<10^{-6}$). This might relate to how the subject attempts to match past and current information.

As was observed in the MIP analysis, $\sum _{M\in \mathcalligra {M}}\Phi _{\mathrm {MIP}}^{M}$ during the stimulus was statistically more significant in the SYNC condition than the ASYNC condition (Welch’s t test: *df* = 13177, t-stat = − 2.3734, $p<0.05$). Although both conditions changed the perception of subject’s body ownership, the $\Phi$ value reflected the difference between the two subjective reports of the participants.

### IIT 2.0 estimates the degree of ownership

In this final subsection, we discuss how IIT 2.0 can estimate subjective reports of body ownership. The original purpose of IIT was to measure the degree of consciousness theoretically. Although the estimation of consciousness itself is still beyond our computational power, this aim could be attained through our small body–brain system consisting of seven time-series of physiological data.

We have thus far observed the average effects of (a)synchronous stimulus on each brain-body system. However, there seem to be individual differences in the subjective strength of RHI: some subjects responded negatively to questionnaire statements Q1–Q3 asking RHI experience (see “Methods” and Table S1) in the SYNC condition, and conversely, some responded positively in the ASYNC condition (Fig. S3). To know why such individual differences emerge, we tested whether individual differences in the experience of the RHI were associated with an index of IIT analysis. First, we averaged the subjective ratings of questions Q1–Q3 for each subject in each condition. This value has been used to represent the overall strength of the illusion, as an RHI index^28^. The RHI index includes the referral of touch over a fake hand rather than purely body ownership. This is appropriate for our purpose because it may express a degree of ABO well, the concurrent feeling of both fake and own hand. We used this index for the following analysis.

**Figure 5:**
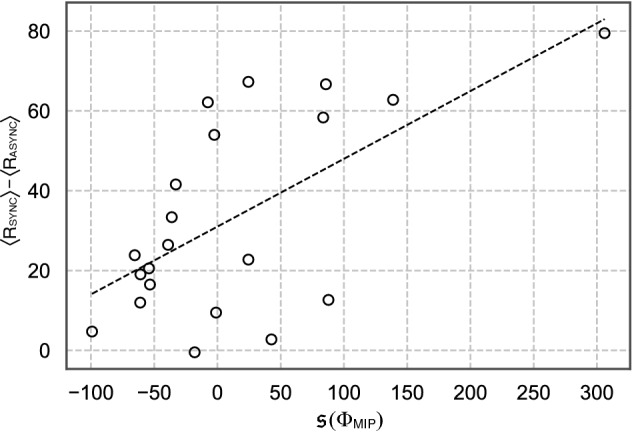
Relationship between the difference in mean subjective reports, $\langle R_{\mathrm {SYNC}} \rangle - \langle R_{\mathrm {ASYNC}} \rangle$, and $\mathfrak {s}(\Phi _{\mathrm {MIP}})$ (see Eq. 1) for each subject (Pearson’s correlation test: $r=0.56$, $n=21$, $p<0.005$; see Table S6 for other parameter settings))

We begin by asking whether the integrated information and $\Phi _{\mathrm {MIP}}$ for each subject can account for individual differences in subjective reports. The answer is no. In both conditions, $\sum _{M\in \mathcalligra {M}}\Phi _{\mathrm {MIP}}^{M}$ (abbr. $\sum \Phi _{\mathrm {MIP}}$) has a low correlation (SYNC: Pearson’s correlation test, $n=21$, $r =0.28$, $p=0.21$. ASYNC: $n=21$, $r =0.20$, $p=0.38$). Interestingly, $\sum \Phi _{\mathrm {MIP}}$ itself did not reflect the subjective reports. In our system, at least, the $\sum \Phi _{\mathrm {MIP}}$ is not the effective estimator of subjective reports.

Next, we calculated the difference in $\sum \Phi _{\mathrm {MIP}}$ between the SYNC and the ASYNC conditions. After subtracting the baseline (mean pre-stimulus phase for each subject), we calculated the difference across stimulus phases and the sum of its time series. Mathematically, this measure can be expressed for each subject *i* as:

1 $$\begin{aligned} \mathfrak {s}_{i}(\Phi _{\mathrm {MIP}})=\sum _{t=300}^{600}\{ \sum _{M\in \mathcalligra {M}_{t}}( \bar{\Phi }_{\mathrm {MIP}}^{M, \mathrm {SYNC}}-\bar{\Phi }_{\mathrm {MIP}}^{M, \mathrm {ASYNC}})\} \end{aligned}$$

where $\bar{\Phi }_{\mathrm {MIP}}^{M, \mathrm {SYNC}}$ means that the integrity of the main complex *M* at time *t* in the SYNC condition subtracted the baseline.

We found that differences in RHI indices between conditions positively correlated with ${s}_{i}(\Phi _{\mathrm {MIP}})$ (Fig. 5; Pearson’s correlation test: $n=21$, $r =0.56$, $p<0.005$). This shows that the $\Phi _{\mathrm {MIP}}$ gaps, rather than of $\Phi _{\mathrm {MIP}}$ itself, can estimate individual differences in subjective rating gaps between experimental conditions.

## Discussion

This study showed that IIT 2.0 could characterise the subjective experience during the rubber hand illusion-that is, ambiguous body ownership (ABO)—in terms of both general tendency and individual difference.

Before summarising the results, let us review the computational meaning of the application of IIT. $\Phi _{\mathrm {MIP}}$s differ from the single or coupled physiological data (e.g., heartbeats, skin conductance, and temperature). These interceptives are sometimes related to the FEP paradigm^20^. However, the relationship between the autonomic system and subjective experience is still unproven (at least at the level of single physiological data). If their relationship should be proven somehow, it would be indirectly.

$\Phi _{\mathrm {MIP}}$ differs from those measures: IIT measures not a change of variables but the change of the system’s interaction structure. $\Phi _{\mathrm {MIP}}$ deals with the interaction heterogeneity of the system rather than its covariance; it measures how entangled information exists inside the system during the stimuli.

Some simple examples are helpful. In complete random interaction systems, $\Phi _{\mathrm {MIP}}$ becomes zero because its mutual information is also zero. In perfectly correlated interaction systems, $\Phi _{\mathrm {MIP}}$ also becomes zero because we cannot discriminate between local and global behaviour. The high $\Phi _{\mathrm {MIP}}$s found in the system have a more heterogeneous interaction structure (for a more detailed comparison, see Median et al.^29^).

$\Phi _{\mathrm {MIP}}$ is a measure positioned within the whole system structure. Therefore, $\Phi _{\mathrm {MIP}}$ can be a more direct measure of the subjective experience of RHI than dealing directly with physiological data. Here we regarded the autonomic and central nervous systems as one system; the results showed that they were complexly intertwined and could not be treated in isolation, even though they vary on different timescales.

We found several insights by applying IIT 2.0 to the small body–brain system. The MIP reveals that the system’s independence emerges during the stimulus phase for both experimental conditions. The location of the MIP cut indicates that the disjunctive parts are not body–brain pairs but intra-brain or intra-body pairs. Before the stimulus, the body–brain system works as one irreducible system. Although the *S* exists even in the stimulus phase, the degree of this irreducibility ($\Phi _{\mathrm {MIP}}^{S}$) decreases. In other words, the system loses the entire entanglement during RHI.

We found several insights by applying IIT 2.0 to the small body—brain system. The MIP revealed that the system’s independence emerged during the stimulus phase in both experimental conditions. The location of the MIP cut indicated that the disjunctive parts were not body–brain pairs but intra-brain or intra-body pairs. Before the stimulus, the body—brain system worked as one irreducible system. Although *S* existed even in the stimulus phase, the degree of this irreducibility ($\Phi _{\mathrm {MIP}}^{S}$) decreased. In other words, the system lost the total entanglement during RHI.

Although the whole system *S* lost its integrity, the subsystem (main complexes) retained high integrity (i.e., $\sum \Phi _{\mathrm {MIP}}$) during the stimulus. The entangled high parts, for example, were {Fz, Cz, RES, and ECG}. As MIP is located at Oz, the complexes, including Fz and Cz, also have a compensated meaning. In the stimulus phase, the brain might raise the integrity for the working memory (Fz) and motor function (Cz) instead of separating the inconsistent visual information (Oz). This relation is logical if we consider that the motor-perception with the subject’s short-term memory (including multisensory integration at the ventral premotor cortex^30,31^) attempts to counterbalance the mismatch in the visual system. Body integrity (RES and EDA) was also observed in the stimulus phase. Notably, ECG (i.e., heartbeat) was excluded from this body integrity (the MIP cut is located at {ECG} in Fig. 2B). This relative independence of the heartbeat might be related to its use as an index of the malleability of body representation^11^.

Although our average IIT analysis explained the general tendencies, individual differences still existed. Some subjects experienced a much stronger feeling of owning the fake hand in the SYNC condition than in the ASYNC condition; but others did not show such a strong difference between conditions. Previous studies have tried to explain these various individual differences from the physiological perspective^21^, but with no consistent conclusions. Here, we found the positive correlation of the $\sum \Phi _{\mathrm {MIP}}$ value with subjective reports as focusing on the differences between conditions (SYNC minus ASYNC). This suggests that subjective evaluation is determined not by a single experience but rather by comparing different experiences. We also attempted the same analysis by applying the peak–end rule, which states that how a subject feels at their peak and at their end determines their judgement of a given experience^32^. The results were the same (Table S6). Furthermore, it is necessary to use time-varying data on the subjective rating to analyse the relationship between the detailed subjective rating (the onset of the illusion) and IIT. This is left for future work.

Finally, we comment on two critical issues on ABO from our analysis:

1. Average tendency of $\Phi _{\mathrm {MIP}}$ variations: We confirmed the graph similarities between the two experimental conditions (note that a statistical difference existed between them). This result might appear somewhat strange, because both conditions generate different phenomenological experiences. However, we should also be aware that the ASYNC condition is not a neutral experience. For instance, some researchers have pointed out that we should not miss that the ASYNC condition generates an “ambiguous sensation” during RHI^33,34^. Furthermore, Perez-Marcos et al. have reported that body image distortion can be elicited by the ASYNC condition but not by the SYNC condition^35^. Their results imply that the ASYNC condition also changes latent body cognition levels. All these results suggest that the ASYNC condition also contribute to change the phenomenological experiences under the radar of the questionnaires.
2. Individual subjective differences and $\Phi _{\mathrm {MIP}}$ s: We confirmed that $\Phi _{\mathrm {MIP}}$ itself was not associated with subjective reports in either condition. Individual differences in RHI experience have been pointed out from several aspects. There are many biases in the subjective reports. First, the anchoring effect is the most famous: past subject ratings influence current ratings^36^. Second, an order effect of the experimental conditions exists^37^. Third, hypothesis awareness also affects subjective ratings^38,39^: the experimental setting changes the subject’s expectations. All these biases show that the experimental setting influences the subjective ratings without the subject’s intention. Another possible reason is perceptual ability^40,41^. For instance, Costantini et al. have shown that the participant’s temporal resolution ability changes the illusion strength in the RHI in the asynchronous condition^42^. Also, proprioceptive accuracy has been shown to associate with RHI strength^43^. Therefore, individual differences in RHI index could be due to a variety of factors; we may need more data to associate with the individual subjective difference from $\Phi _{\mathrm {MIP}}$ itself.
  While there was no association between the RHI index and $\Phi _{\mathrm {MIP}}$ in either condition, we found that the difference in $\Phi _{\mathrm {MIP}}$ between conditions (i.e., $\mathfrak {s}(\Phi _{\mathrm {MIP}})$) correlated with the difference in RHI indices between conditions, suggesting that $\Phi _{\mathrm {MIP}}$ reflects individual differences in RHI experience when comparing conditions. So what component of the RHI index is crucial for association with $\mathfrak {s}(\Phi _{\mathrm {MIP}})$?
  The difference in Q3 between conditions (dQ*x*, where *x* represents an item number) was correlated significantly with $\mathfrak {s}(\Phi _{\mathrm {MIP}})$, and its coefficient was slightly smaller than the RHI index (Pearson’s correlation test: $n = 21$, $r = 0.53$, $p < 0.05$). The dQ2 was also correlated significantly, and its coefficient was even smaller than that of dQ3 ($n = 21$, $r = 0.44$, $p < 0.05$). There was no significant correlation between dQ1 and $\mathfrak {s}(\Phi _{\mathrm {MIP}})$ ($n = 21$, $r = 0.40$, $p = 0.07$). The RHI index can be classified into “ownership” (Q3) and “referral of touch” (Q1 and Q2)^28^. Therefore, these results of correlation analysis suggest that $\mathfrak {s}(\Phi _{\mathrm {MIP}})$ reflects “ownership” rather than “referral of touch”. In this sense, perhaps “ownership” distorts the “referral touch”^44^.
  However, it might be too early to assume that $\mathfrak {s}(\Phi _{\mathrm {MIP}})$ represents pure ownership. We found significant differences between conditions even in the control questions Q7 and Q8 (paired t-test, $p < 0.05$). Interestingly, dQ7 and dQ8 ratings were significantly correlated with dQ3 (i.e., ownership) (Pearson’s correlation test, dQ7: $n = 21$, $r = 0.67$, $p < 0.005$; dQ8: $n = 21$, $r = 0.45$, $p < 0.05$). Calculating mean values of dQ1, dQ2, dQ3, dQ7, and dQ8 for each participant, we found that these values were correlated significantly with $\mathfrak {s}(\Phi _{\mathrm {MIP}})$ and, importantly, had larger coefficients than the RHI index (Pearson’s correlation test: $n = 21$, $r = 0.59$, $p < 0.005$).
  How should we consider this stronger correlation caused by adding control questions to the RHI index? We should note that the participants gave negative answers (50) to Q7 and Q8 in almost all conditions (Q7: mean 57.3, SD 31.8 for SYNC; mean 43.8, SD 29.2 for ASYNC; Q8: mean 37.3, SD 27.7 for SYNC; mean 28.7, SD 26.0 for ASYNC). Remarkably, these ratings indicated that denial belief was weakened in the SYNC condition. The significant correlation of dQ3 with dQ7 and dQ8 indicated that participants who felt ownership of the dummy (maybe unconsciously) tended to give a neutral answer rather than a negative one to these questions. Q7 and Q8 were related to the physical properties and location, respectively, of the dummy hand. Participants might have had an ambiguous feeling of “It’s both my hand and a dummy hand.” Thus, we assumed that $\mathfrak {s}(\Phi _{\mathrm {MIP}})$ measured the ambiguous sensation accompanying ownership, ABO, rather than ownership itself.

As mentioned in the Introduction, $\Phi _{\mathrm {MIP}}$ in IIT is used to measure the gap between feeling and judgement. Given these considerations, it may be correct to consider that what $\Phi _{\mathrm {MIP}}$ measures is not a “concrete” phenomenological state but rather a vague sensation that includes positive ratings (such as referral touch and ownership) and negative ratings observed in control questions, that is, ABO. In other words, ABO reflects entangled information for the single brain–body system.

## Methods

### Basic concept of IIT

Let us first explain IIT concepts for the sake of readers unfamiliar with IIT. Although many concepts exist in IIT, we focus on three: minimum information partition (MIP), main complexes, and integrated information $\Phi$.

IIT deals with the intrinsic, rather than extrinsic, information; it only depends on inner variables. Typical information theories used in biology focus on the relation between external inputs and their results. In this setting, the system itself becomes a black box. The aim of IIT is to consider this black box in terms of “what the system is” rather than “what the system does”^45^. The basic concept of IIT expresses mutual information between the past and current system states; mathematically, this is expressed as $I(X(t-\tau );X(t))$.

However, this simple mutual information contains redundant information on the system’s integrity, which should be measured as the system’s irreducibility to its parts (subsystems). The information obtained from the subsystems must be subtracted from the entire information $I(X(t-\tau );X(t))$. The rest of the information represents the system’s integrity; therefore, the problem for IIT lies in the determination of the system partitions.

This study applies IIT as the mismatch decoding proposed by Oizumi et al.^18^ because $\Phi ^{*}$ satisfies the following properties. (i) Non-negativity: the lower bound of $\Phi ^{*}$ is zero; (ii) sensitivity to noise correlation: $\Phi ^{*}$ deals with external correlated noise^29^; and (iii) applicability to continuous variables. Sometimes, the IIT as the mismatch decoding is classified as “IIT 2.0” to distinguish it from IIT 3.0^46–48^. In this paper, we did not apply IIT 3.0 to the physiological data because IIT 3.0 deals only with discrete values.

#### Integrated information ($\Phi ^{*}$) and minimum information partition (MIP)

As mentioned in the previous section, the mode of partitioning is essential to determining the system’s integrity. MIP is the system partition where the system’s integrity is at its minimum. The system’s integrity in $\Phi ^{*}$ is given by

2 $$\begin{aligned} \Phi ^{*} = I(X(t-\tau );X(t)) - I^{*}[X; \tau ,\mathcalligra {P}_{S}]\,, \end{aligned}$$

where $I(X(t-\tau );X(t))$ is the mutual information between the current *X*(*t*) and past states $X(t-\tau )$; *S* is the set of all nodes of a given system; $\mathcal {P}_{S}$ is the set of all bi-partitions (total $2^{|S|}-1$ partitions); $I^{*}(X; \tau ,\mathcal {P}_{S})$ is a “hypothetical” mutual information, indicating the mismatched decoding in the partitioned probability distribution. More precisely, $I^{*}(X; \tau ,\mathcalligra {P}_{S})$ is given as the partition $\max _{\beta }\tilde{I}(\beta ;X, \tau ,\mathcalligra {P}_{S})$ that minimises $\Phi ^{*}$ (see listed studies^18,29^ for further details about this expression).

Because $\Phi ^{*}$ depends on the partition $\pi (\in \mathcalligra {P}_{S})$, MIP is the partition that minimises the integrated information.

3 $$\begin{aligned} \pi _{\mathrm {MIP}} := \arg \min _{\pi \in\mathcalligra {P}_{S}} \Phi ^{*}(\pi ) \end{aligned}$$

The integrated information of $\pi _{\mathrm {MIP}}$ is expressed as $\Phi ^{*}(\pi _{\mathrm {MIP}})$. We simply denote $\Phi ^{*}(\pi _{\mathrm {MIP}})$ as $\Phi _{\mathrm {MIP}}$. Notably, if $\Phi _{\mathrm {MIP}}$ equals zero, the parts of the system are mutually independent; that is, there is no interaction between the parts. In this sense, $\Phi _{\mathrm {MIP}}$ characterises the system’s irreducibility to its parts.

#### Main complexes

Generally, we can compute $\Phi _{\mathrm {MIP}}$ for any subsystem in the system, not only for the set *S*. We denote each $\Phi _{\mathrm {MIP}}$ for a subsystem *T* as $\Phi _{\mathrm {MIP}}^{T}$, where $T\subset S$. A “complex” is a subsystem $C (\subset S)$, where $\Phi _{\mathrm {MIP}}^{C} > \Phi _{\mathrm {MIP}}^{T}$ for all supersets of *S*. From this definition, we can define the main complexes as those with the local maximum $\Phi _{\mathrm {MIP}}^{M}$.

*Definition (main complex)*: A main complex is a complex *M* satisfying $\Phi _{\mathrm {MIP}}^{M} > \Phi _{\mathrm {MIP}}^{R}$ for any subsystem $R \subset M$.

The definition says that if two main complexes exist (say, *A* and *B*), then they are exclusive. IIT researchers consider these main complexes to be the system’s information core that might be related to our conscious experience. The validity of this assumption remains to be demonstrated, although there is no doubt that such information cores play a vital role in living systems^49–51^.

### Application to RHI

#### Participants

Twenty-two healthy male adults (mean age, 21.1 years; SD, 0.5) were recruited from among the students at Nagaoka University of Technology. All participants gave written consent to participate after receiving an explanation of the procedures involved. The study was conducted according to the principles of the Declaration of Helsinki and was approved by the Ethics Committee of Nagaoka University of Technology.

#### Procedure

Participants sat on a chair with their left arm on a table with the palm facing down. The experimenter stood opposite the participant across the table. A realistic-looking left rubber hand was placed to the right of the participant’s real left hand. The distance between the index finger of the rubber hand and that of the real hand was 15 cm. Two grey towels covered the space from the participant’s shoulder to the participant’s wrist and the proximal end of the artificial hand. A 45 cm $\times$ 60 cm standing screen between the rubber hand and the real hand prevented participants from viewing their own left hand (see Fig. S1).

Two experimental conditions were investigated in our study: synchronous and asynchronous stroking. The experiments comprised two blocks corresponding to these conditions. The sequence of the blocks was randomised and counterbalanced across participants. Each block lasted for 15 min, with a few minutes of inter-block intervals: 5-min rest periods (Rest Phase: Pre and Post) before and after a 5-min stroking period (Stimulus Phase). The rubber hand and unseen hand were stroked by two brushes synchronously or asynchronously during the stroking period. A trained experimenter indicated the predetermined pattern and frequency (40 bpm) of stimulation using a metronome through an earphone. In the asynchronous condition, the dummy hand was touched immediately after touching the hidden real hand by the metronome; that is, every visual stimulus followed a tactile stimulus in the asynchronous condition. Thus, the tactile stimulation was identical for both conditions. After the second rest period, the participants completed a questionnaire about their subjective experiences during the stroking period.

#### Measurement

##### Physiological measurement

Throughout each experimental block, EEG, electrocardiogram (ECG), respiration airflow (RES), and electrodermal activity (EDA) were recorded using a bio-amplifier (Polymate Pro MP 6000, Miyuki Giken Corp., Tokyo, Japan) at a sampling rate of 250 Hz and with a 24-bit resolution, using a 50-Hz notch filter. In particular, for the EEG, four signals (Fz, Cz, Pz, Oz) were measured at the locations of the four electrodes on the scalp according to the international 10-20 system for measuring the four EEG signals (Fz, Cz, Pz, Oz); here, we used a high-frequency 50 Hz filter and a low-frequency 1 Hz filter.

##### Questionnaire

The questionnaire comprised nine statements from the original RHI study^1^ presented on a computer screen in random order (see Table S1). Participants indicated their responses by clicking on a visual-analogue scale ranging from strong agreement (100) to strong disagreement (0), including neither agreement nor disagreement (50). All the questions and instructions were presented in Japanese. Questionnaire data from one participant were excluded from the analysis because they were lost owing to mechanical issues.

#### Data setting for IIT 2.0 application

Oizumi et al. proposed approximation methods for the computational problem of IIT^18,19,52,53^. This study applied their “Practical $\Phi$ Toolbox for MATLAB” to the physiological data; we considered only seven types of physiological data for the 22 subjects. The exhaustive method (computing all possible MIPs) can be applied in realistic computation time for such a small system.

Another parameter is time delay $\tau$ (IIT has only two constraints: partition $\pi$ and time delay $\tau$). To choose the suitable parameter, we computed $\Phi _{\mathrm {MIP}}$ for $\tau$ from 0 to 250 frames. The time delay $\tau$ is the point where the mean is the maximum $\Phi _{\mathrm {MIP}}$ for all subjects. We found that this $\tau$ is approximately 50 frames (i.e. 0.2 s). This value seems to be suitable because the value is the same as the human response time (Fig. 2S).

The time series of each set of raw data was 225,000 frames (250 frames per second). Because we applied a 50-frame delay (0.2 s) for the computation of IIT 2.0, sufficient data series for IIT 2.0 are required. We attempted 250 frames (1 s), 500 frames (2 s), and 750 frames (4 s) for IIT 2.0 computations—the time window shifts 250 frames each. The first set of blank data for some frames (250, 500, 750 frames) was set at zero (only the first three steps at most for 750 frames), which sum up to 900 datasets for each subject. The results for all frame samples were almost the same (Tables S2–S6).

We extracted all measures related to integrated information for 900 steps (1 s for one step). The first third ($0{-}300$ steps) and the last third ($600{-}900$ steps) correspond to the pre- and post-stimulus resting states. The middle section ($300{-}600$ steps) is where the synchronous or asynchronous stimulus was applied. For all statistical tests, we detrended the obtained $\Phi$ time series because our focus was on the variation due to the stimulus for our rubber hand experiments. The negative value of $\Phi _{\mathrm {MIP}}$ is due to this treatment.

## Supplementary Information

Supplementary Information.

## Data Availability

All data are available from the corresponding author upon reasonable request.
