# Supplementary material for: The ambiguous feeling between “mine” and “not-mine” measured by integrated information theory during rubber hand illusion
Source: Sci Rep. 2022 Oct 26;12:18002. doi: 10.1038/s41598-022-22927-1 (PMC9606129; doi:10.1038/s41598-022-22927-1)
Supplement: Supplementary file 1 — Supplementary Information. [file 41598_2022_22927_MOESM1_ESM.pdf]

Supplementary Information to
"The Ambiguous Feeling between "Mine" and "Not-Mine" Measured by Integrated Information Theory During Rubber Hand Illusion"

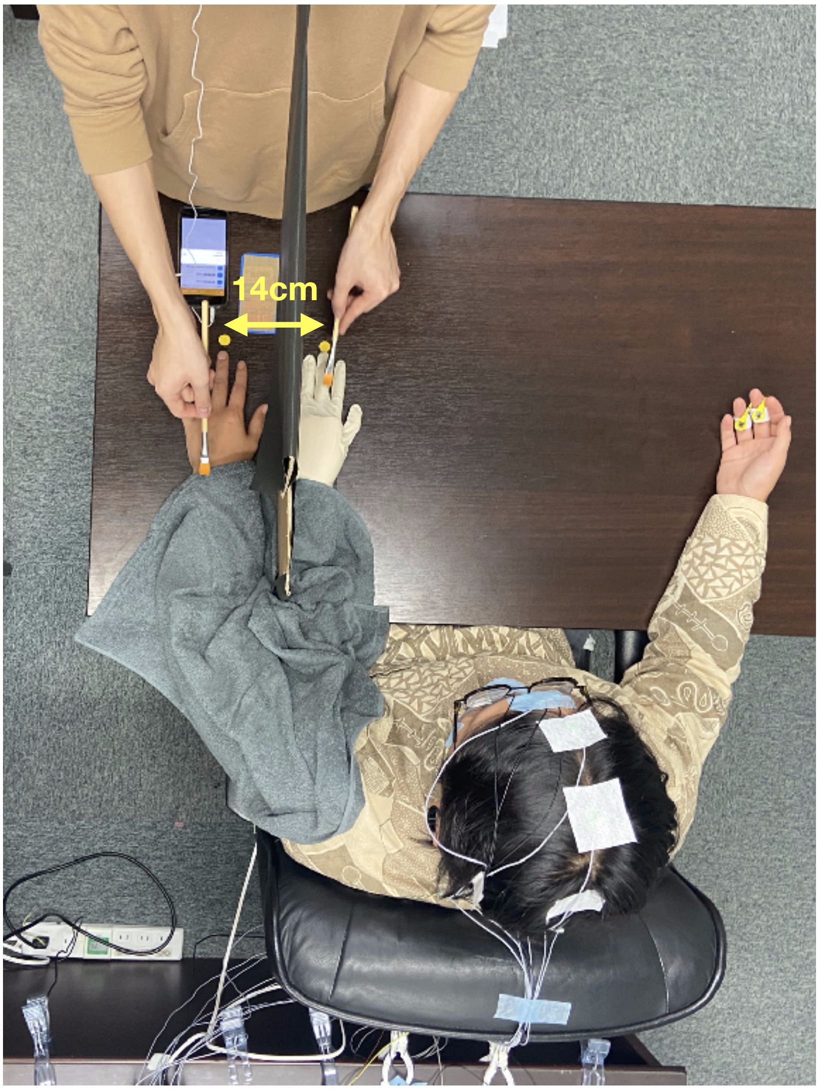

Fig. S1. Experimental setup in the stimulus phase. A dummy left hand was placed in front of the participant. The participant's left hand was hidden. The right hand, with a probe to measure skin conductance (EDA), rested on the table. EEG electrodes were installed on the participant's scalp. In addition, participants were equipped with ECG electrodes and nostril cannulae to measure their respiration flow.

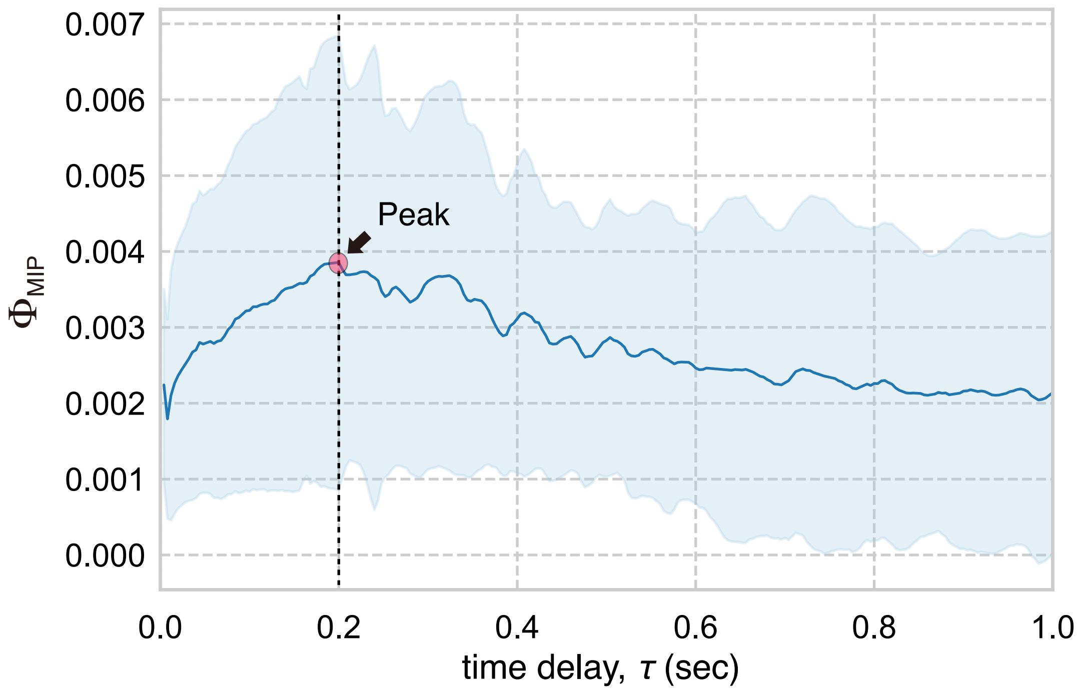

Fig. S2. Φ_{MIP} values along time delay τ for the entire system S. The frame size is 900 s. The Φ_{MIP} peak is located at 0.2 s.

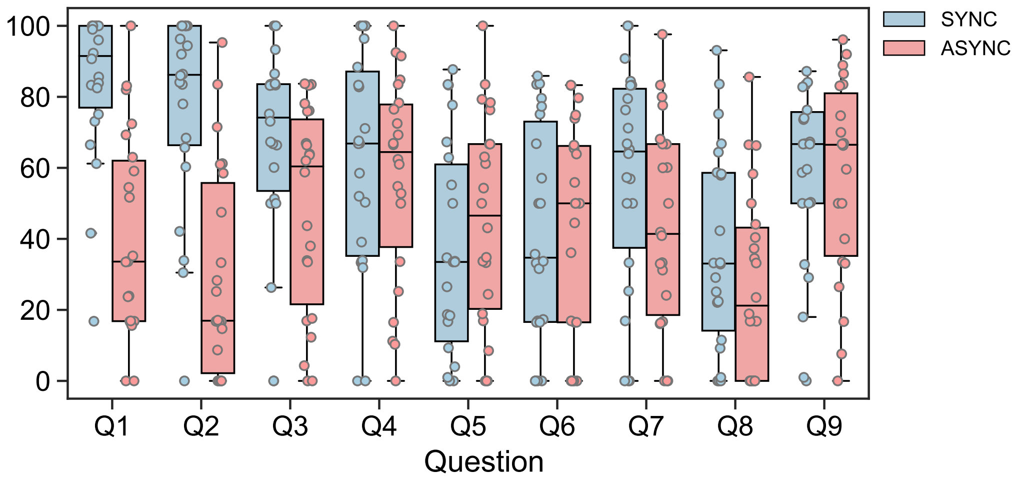

Fig S3. Boxplot of rating scores for each questionnaire item. The data are presented in Table S1.

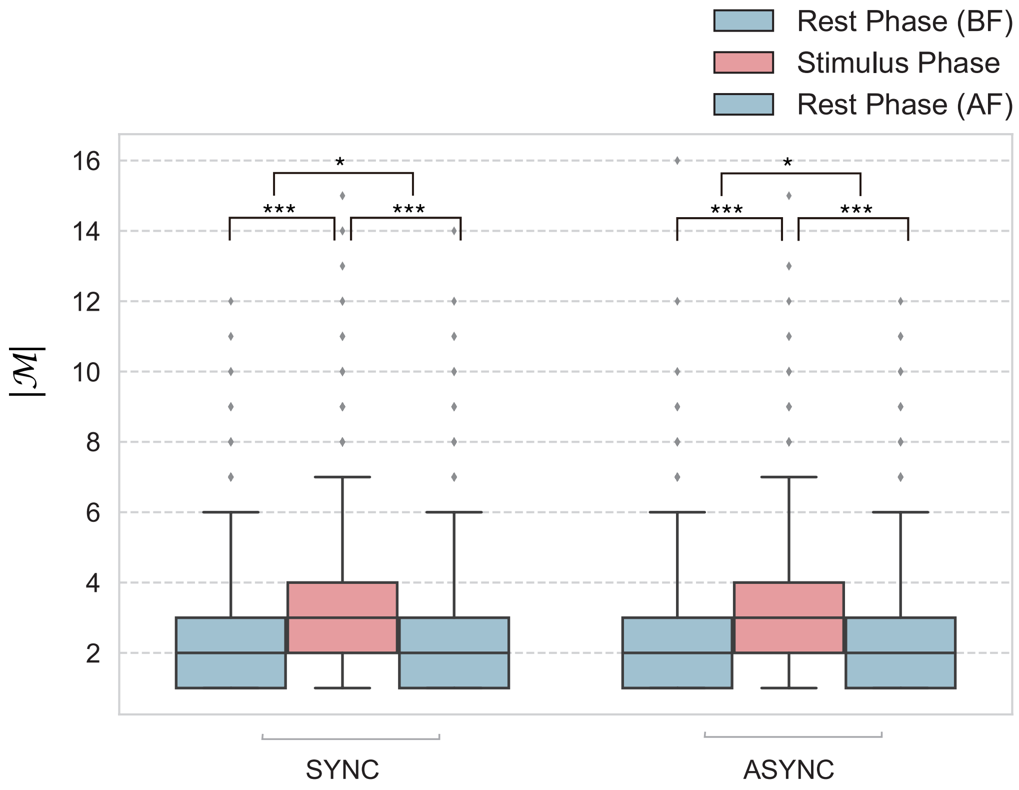

Fig. S4. Boxplot of the number of complexes for each main complex. The tendency is the same as the Φ^{M}_{MIP} ($M \in \mathcal{M}$) distribution in Figure 4.

S1 Table. Questionnaire items[1].

Index	Contents
**Q1**	It seemed as if I felt the touch of a paintbrush in the location where I saw the dummy hand touched.
**Q2**	It seemed as if the touch I felt was caused by the paintbrush stroking the dummy hand.
**Q3**	I felt as if the dummy hand was my hand.
**Q4**	I felt as if my real hand was drifting towards the dummy hand.
**Q5**	It seemed as if I might have more than one left hand.
**Q6**	It seemed as if the touch I felt emanated from somewhere between my real hand and the dummy hand.
**Q7**	I felt as if my real hand was evolving into the dummy hand.
**Q8**	It appeared as if the dummy hand was drifting towards my real hand.
**Q9**	The dummy hand appeared to resemble my real hand visually.

1. Botvinick, M. & Cohen, J. Rubber hands 'feel' touch that eyes see. Nature 391, 756–756, DOI: 10.1038/35784 (1998).

S2 Table. Statistical tests of Figure 1.

T=250

type	t-stat	df	p_value
**SYNC:** Pre-- Stimulus	25.9318	1.29E+04	**1.40E-144**
**SYNC**: Post--Stimulus	26.7502	1.29E+04	**1.76E-153**
**SYNC**: Pre--Post	0.7135	1.29E+04	0.4756
**ASYNC:** Pre-- Stimulus	26.5283	1.29E+04	**4.90E-151**
**ASYNC**: Post--Stimulus	26.8538	1.30E+04	**1.19E-154**
**ASYNC**: Pre--Post	0.1513	1.32E+04	0.8798

type	t-stat	df	p_value
**SYNC-ASYNC:** Stimulus	2.2561	1.32E+04	**2.41E-02**

T=500

type	t-stat	df	p_value
**SYNC:** Pre-- Stimulus	19.8788	1.29E+04	**1.15E-86**
**SYNC**: Post--Stimulus	21.0739	1.29E+04	**5.42E-97**
**SYNC**: Pre--Post	0.5795	1.32E+04	0.5622
**ASYNC:** Pre-- Stimulus	19.9379	1.29E+04	**3.64E-87**
**ASYNC**: Post--Stimulus	21.083	1.30E+04	**4.50E-97**
**ASYNC**: Pre--Post	1.1423	1.32E+04	0.2534

type	t-stat	df	p_value
**SYNC-ASYNC:** Stimulus	0.2264	1.32E+04	8.21E-01

T=750

type	t-stat	df	p_value
**SYNC:** Pre-- Stimulus	17.6617	1.28E+04	**5.14E-69**
**SYNC**: Post--Stimulus	18.4013	1.29E+04	**1.10E-74**
**SYNC**: Pre--Post	0.1331	1.32E+04	0.8941
**ASYNC:** Pre-- Stimulus	17.3597	1.28E+04	**9.17E-67**
**ASYNC**: Post--Stimulus	18.6736	1.30E+04	**7.92E-77**
**ASYNC**: Pre--Post	1.0263	1.32E+04	0.3048

type	t-stat	df	p_value
**SYNC-ASYNC:** Stimulus	-0.2248	1.32E+04	8.22E-01

S3 Table. Statistical tests of Figure 2.

{Oz}-cut : T=250

type	t-stat	df	p_value
**SYNC:** Pre-- Stimulus	18.4226	3.84E+03	**1.08E-72**
**SYNC**: Post--Stimulus	19.4833	4.41E+03	**3.60E-81**
**SYNC**: Pre--Post	0.4069	4.31E+03	0.6841
**ASYNC:** Pre-- Stimulus	17.7124	3.38E+03	**3.34E-67**
**ASYNC**: Post--Stimulus	18.9255	3.99E+03	**1.45E-76**
**ASYNC**: Pre--Post	-0.0951	3.86E+03	0.9243

{Oz}-cut : T=500

type	t-stat	df	p_value
**SYNC:** Pre-- Stimulus	39.7054	3.99E+03	**7.16E-291**
**SYNC**: Post--Stimulus	44.7446	4.19E+03	**0.00E+00**
**SYNC**: Pre--Post	-3.3416	3.12E+03	**8.43E-04**
**ASYNC:** Pre-- Stimulus	48.9736	4.63E+03	**0.00E+00**
**ASYNC**: Post--Stimulus	57.5847	4.90E+03	**0.00E+00**
**ASYNC**: Pre--Post	-4.3831	2.88E+03	**1.21E-05**

{Oz}-cut : T=750

type	t-stat	df	p_value
**SYNC:** Pre-- Stimulus	56.3785	4.73E+03	**0.00E+00**
**SYNC**: Post--Stimulus	63.8736	4.96E+03	**0.00E+00**
**SYNC**: Pre--Post	2.176	2.21E+03	**0.0297**
**ASYNC:** Pre-- Stimulus	34.3748	3.83E+03	**6.06E-226**
**ASYNC**: Post--Stimulus	42.9057	4.10E+03	**0.00E+00**
**ASYNC**: Pre--Post	5.2859	2.10E+03	**1.38E-07**

{Fz}-cut : T=250

type	t-stat	df	p_value
**SYNC:** Pre-- Stimulus	12.3052	4.36E+03	**3.12E-34**
**SYNC**: Post--Stimulus	12.0027	4.16E+03	**1.18E-32**
**SYNC**: Pre--Post	-0.1405	4.78E+03	0.8883
**ASYNC:** Pre-- Stimulus	13.3828	4.11E+03	**5.18E-40**
**ASYNC**: Post--Stimulus	13.0455	4.00E+03	**4.01E-38**
**ASYNC**: Pre--Post	-0.5492	4.96E+03	0.5829

{Fz}-cut : T=500

type	t-stat	df	p_value
**SYNC:** Pre-- Stimulus	19.4746	7.25E+02	**2.89E-68**
**SYNC**: Post--Stimulus	20.175	7.06E+02	**8.05E-72**
**SYNC**: Pre--Post	0.7289	9.61E+02	0.4663
**ASYNC:** Pre-- Stimulus	17.3405	8.10E+02	**1.59E-57**
**ASYNC**: Post--Stimulus	18.8085	7.76E+02	**2.60E-65**
**ASYNC**: Pre--Post	-2.6152	1.04E+03	**0.009**

{Fz}-cut : T=750

type	t-stat	df	p_value
**SYNC:** Pre-- Stimulus	16.833	6.61E+02	**3.52E-53**
**SYNC**: Post--Stimulus	17.3158	6.33E+02	**2.80E-55**
**SYNC**: Pre--Post	-1.8546	8.82E+02	0.064
**ASYNC:** Pre-- Stimulus	14.386	7.59E+02	**1.13E-41**
**ASYNC**: Post--Stimulus	11.322	7.51E+02	**1.51E-27**
**ASYNC**: Pre--Post	1.7863	9.38E+02	0.0744

{ECG}-cut : T=250

type	t-stat	df	p_value
**SYNC:** Pre-- Stimulus	5.4314	6.14E+02	**8.07E-08**
**SYNC**: Post--Stimulus	6.5118	6.39E+02	**1.50E-10**
**SYNC**: Pre--Post	1.2559	1.09E+03	0.2094
**ASYNC:** Pre-- Stimulus	5.3576	6.59E+02	**1.17E-07**
**ASYNC**: Post--Stimulus	6.119	7.24E+02	**1.54E-09**
**ASYNC**: Pre--Post	1.2149	1.13E+03	0.2247

{ECG}-cut : T=500

type	t-stat	df	p_value
**SYNC:** Pre-- Stimulus	44.2334	5.26E+03	**0.00E+00**
**SYNC**: Post--Stimulus	45.0654	5.06E+03	**6.4558e-319**
**SYNC**: Pre--Post	-1.1181	6.29E+03	0.2636
**ASYNC:** Pre-- Stimulus	47.5725	4.98E+03	**0.00E+00**
**ASYNC**: Post--Stimulus	50.4526	5.22E+03	**0.00E+00**
**ASYNC**: Pre--Post	-0.0745	6.40E+03	0.9406

{ECG}-cut : T=750

type	t-stat	df	p_value
**SYNC:** Pre-- Stimulus	41.6206	6.14E+03	**0.00E+00**
**SYNC**: Post--Stimulus	40.6452	5.94E+03	**6.4558e-319**
**SYNC**: Pre--Post	0.8253	7.43E+03	0.4092
**ASYNC:** Pre-- Stimulus	49.5497	6.19E+03	**0.00E+00**
**ASYNC**: Post--Stimulus	46.2168	5.88E+03	**0.00E+00**
**ASYNC**: Pre--Post	-2.6603	7.50E+03	**0.0078**

S4 Table. Statistical tests of Figure 3.

T=250

type	t-stat	df	p_value
**SYNC:** Pre-- Stimulus	-23.7818	1.24E+04	**2.82E-122**
**SYNC**: Post--Stimulus	-21.8958	1.24E+04	**2.65E-104**
**SYNC**: Pre--Post	-2.093	1.32E+04	**0.0364**
**ASYNC:** Pre-- Stimulus	-22.0088	1.24E+04	**2.45E-105**
**ASYNC**: Post--Stimulus	-18.8627	1.30E+04	**2.54E-78**
**ASYNC**: Pre--Post	-2.0867	1.29E+04	**0.0369**

type	t-stat	df	p_value
**SYNC-ASYNC:** Stimulus	-2.3734	1.32E+04	**1.76E-02**

T=500

type	t-stat	df	p_value
**SYNC:** Pre-- Stimulus	-17.2215	1.32E+04	**9.57E-66**
**SYNC**: Post--Stimulus	-14.4425	1.32E+04	**6.37E-47**
**SYNC**: Pre--Post	-2.7914	1.32E+04	**0.0053**
**ASYNC:** Pre-- Stimulus	-14.8753	1.32E+04	**1.20E-49**
**ASYNC**: Post--Stimulus	-12.1782	1.32E+04	**6.18E-34**
**ASYNC**: Pre--Post	-2.3273	1.32E+04	**0.02**

type	t-stat	df	p_value
**SYNC-ASYNC:** Stimulus	2.7035	1.32E+04	**6.90E-03**

T=750

type	t-stat	df	p_value
**SYNC:** Pre-- Stimulus	-9.145	1.32E+04	**6.82E-20**
**SYNC**: Post--Stimulus	-7.267	1.32E+04	**3.88E-13**
**SYNC**: Pre--Post	-1.85	1.32E+04	0.0643
**ASYNC:** Pre-- Stimulus	-5.8778	1.32E+04	**4.26E-09**
**ASYNC**: Post--Stimulus	-3.4378	1.32E+04	**5.88E-04**
**ASYNC**: Pre--Post	-2.2432	1.32E+04	**0.0249**

type	t-stat	df	p_value
**SYNC-ASYNC:** Stimulus	3.4274	1.32E+04	**6.11E-04**

S5 Table. Statistical tests of Figure 4.

M={EDA, RES}: T=250

type	t-stat	df	p_value
**SYNC:** Pre-- Stimulus	-15.1585	5.52E+03	**6.96E-51**
**SYNC**: Post--Stimulus	-14.5451	5.49E+03	**4.65E-47**
**SYNC**: Pre--Post	-0.8348	5.18E+03	0.4039
**ASYNC**: Pre-- Stimulus	-15.0072	5.32E+03	**6.82E-50**
**ASYNC**: Post--Stimulus	-13.0683	5.46E+03	**1.88E-38**
**ASYNC**: Pre--Post	-1.3898	4.99E+03	0.1646

M=S : T=250

type	t-stat	df	p_value
**SYNC:** Pre-- Stimulus	-0.4404	1.77E+02	6.60E-01
**SYNC**: Post--Stimulus	-0.9047	1.73E+02	3.67E-01
**SYNC**: Pre--Post	0.5034	5.28E+02	0.6149
**ASYNC**: Pre-- Stimulus	1.236	1.67E+02	2.18E-01
**ASYNC**: Post--Stimulus	1.2067	1.84E+02	2.29E-01
**ASYNC**: Pre--Post	-0.0081	5.72E+02	0.9936

{ECG}-cut : T=250

type	t-stat	df	p_value
**SYNC:** Pre-- Stimulus	-0.6204	9.63E+01	5.37E-01
**SYNC**: Post--Stimulus	0.3641	9.70E+01	7.17E-01
**SYNC**: Pre--Post	-1.5571	4.44E+02	0.1202
**ASYNC**: Pre-- Stimulus	1.8865	1.40E+02	6.13E-02
**ASYNC**: Post--Stimulus	1.2291	1.47E+02	2.21E-01
**ASYNC**: Pre--Post	0.8352	4.75E+02	0.404

M={EDA, RES}: T=500

type	t-stat	df	p_value
**SYNC:** Pre-- Stimulus	-11.5543	7.85E+03	**1.24E-30**
**SYNC**: Post--Stimulus	-9.8393	7.79E+03	**1.03E-22**
**SYNC**: Pre--Post	-1.5289	7.44E+03	0.1263
**ASYNC**: Pre-- Stimulus	-10.6081	7.55E+03	**4.17E-26**
**ASYNC**: Post--Stimulus	-8.4315	7.41E+03	**4.06E-17**
**ASYNC**: Pre--Post	-1.766	7.25E+03	0.0774

M={Cz, Pz} : T=500

type	t-stat	df	p_value
**SYNC:** Pre-- Stimulus	-4.0939	1.47E+02	**6.97E-05**
**SYNC**: Post--Stimulus	-2.897	1.28E+02	**4.40E-03**
**SYNC**: Pre--Post	-1.5105	2.55E+02	0.1321
**ASYNC**: Pre-- Stimulus	-3.2031	1.36E+02	**1.70E-03**
**ASYNC**: Post--Stimulus	-1.9104	1.35E+02	5.82E-02
**ASYNC**: Pre--Post	-2.3614	2.57E+02	**0.019**

{ECG}-cut : T=500

type	t-stat	df	p_value
**SYNC:** Pre-- Stimulus	-0.6901	2.14E+02	4.91E-01
**SYNC**: Post--Stimulus	0.0036	1.68E+02	9.97E-01
**SYNC**: Pre--Post	-0.9286	2.94E+02	0.3539
**ASYNC**: Pre-- Stimulus	-1.4355	2.36E+02	1.53E-01
**ASYNC**: Post--Stimulus	-0.2628	2.22E+02	7.93E-01
**ASYNC**: Pre--Post	-1.6096	3.40E+02	0.1084

M={EDA, RES}: T=750

type	t-stat	df	p_value
**SYNC:** Pre-- Stimulus	-5.0646	8.82E+03	**4.17E-07**
**SYNC**: Post--Stimulus	-4.199	8.65E+03	**2.71E-05**
**SYNC**: Pre--Post	-0.7176	8.26E+03	0.473
**ASYNC**: Pre-- Stimulus	-2.0764	8.48E+03	**3.79E-02**
**ASYNC**: Post--Stimulus	-0.7841	8.41E+03	4.33E-01
**ASYNC**: Pre--Post	-1.2094	8.05E+03	0.2265

M={Cz, Pz} : T=750

type	t-stat	df	p_value
**SYNC:** Pre-- Stimulus	-4.8691	1.78E+02	**2.46E-06**
**SYNC**: Post--Stimulus	-2.7935	1.79E+02	**5.80E-03**
**SYNC**: Pre--Post	-3.1548	3.23E+02	**0.0018**
**ASYNC**: Pre-- Stimulus	-2.5377	1.90E+02	**1.20E-02**
**ASYNC**: Post--Stimulus	-2.1693	1.89E+02	**3.13E-02**
**ASYNC**: Pre--Post	-0.5484	3.29E+02	0.5838

{Oz, EDA}-cut : T=750

type	t-stat	df	p_value
**SYNC:** Pre-- Stimulus	0.7691	1.29E+02	4.43E-01
**SYNC**: Post--Stimulus	0.304	5.60E+01	7.62E-01
**SYNC**: Pre--Post	2.4224	1.83E+02	**0.0164**
**ASYNC**: Pre-- Stimulus	1.118	1.56E+02	2.65E-01
**ASYNC**: Post--Stimulus	0.986	1.21E+02	3.26E-01
**ASYNC**: Pre--Post	0.3349	2.75E+02	0.7379

S6 Table. Statistical tests of Figure 5.

Figure 5

Time	correlation coefficient	p value
T=250	0.56056	**0.0082112**
T=500	0.57694	**0.0061781**
T=750	0.53638	**0.012187**

Peak-End Law in Figure 5

Time	correlation coefficient	p value
T=250	0.5473	**0.0102**
T=500	0.4872	**0.0251**
T=750	0.4482	**0.0416**

SYNC only

Time	correlation coefficient	p value
T=250	0.2846	0.2112
T=500	0.0919	0.6919
T=750	-0.0578	0.8036

ASYNC only

Time	correlation coefficient	p value
T=250	0.2035	0.3763
T=500	0.2275	0.3212
T=750	0.1289	0.5775
